# GW Approximation Coupled with Classical Fluctuating Charges and Dipoles

**DOI:** 10.1021/acs.jctc.5c01722

**Published:** 2025-12-23

**Authors:** Giovanni Nottoli, Piero Lafiosca, Frank Ernesto Quintela Rodríguez, Franco Egidi, Arno Förster, Chiara Cappelli

**Affiliations:** † 226478 Scuola Normale Superiore, Piazza dei Cavalieri 7, Pisa 56126, Italy; ‡ 19004 Software for Chemistry and Materials NV, De Boelelaan 1109, Amsterdam 1081HV, The Netherlands; § 1190 Vrije Universiteit Amsterdam, De Boelelaan 1105, Amsterdam 1081HV, The Netherlands

## Abstract

We propose a novel multiscale QM/classical methodology based on the GW approximation combined with the fluctuating charges (FQ) and fluctuating charges and dipoles (FQFμ) force fields. The GW approximation is exploited to capture electron correlation effects, while FQ or FQFμ is used to model the mutual polarization effects between the quantum GW system and its surrounding environment in a multiscale fashion. The model is validated through test calculations of ionization potentials of aqueous phenol and applied to the Green Fluorescent Protein (GFP) chromophore (4-hydroxybenzylidene-1,2-dimethylimidazolinone—p-HDBI) in aqueous solution.

## Graphical abstract

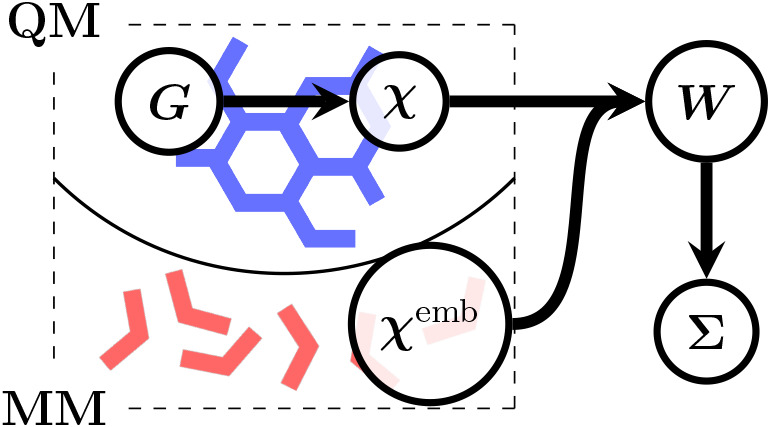

## 1 Introduction

Describing the interaction between a molecule and its environment, which can greatly influence the molecule’s electronic structure, energy levels, and response to an external electric field,^1^ presents significant challenges in quantum chemistry. These effects are particularly relevant in systems where the environment—whether it be a solvent,^2^ a protein matrix,^3^ or a solid-state structure^4^—plays a critical role in determining molecular behavior. For instance, in biological systems, the local environment surrounding a molecule, such as a chromophore, can markedly alter its spectroscopic properties,^5^ while in materials science, the surrounding matrix can significantly impact the charge transport properties of organic semiconductors.^6^ A thorough understanding of these environmental effects is essential for designing molecules with tailored functionalities, optimizing chemical reactions, and accurately interpreting experimental observations.

The goal of this work is to develop a computationally efficient method that enables the evaluation of molecular properties under the influence of an external environment. The challenge lies in balancing accuracy and computational cost: the method must capture the electronic correlation necessary for a reliable description of the molecule’s electronic structure^7,8^ while also efficiently accounting for environmental effects without significantly increasing computational resources. This balance is essential for studying large or complex systems, where traditional quantum mechanical methods would be prohibitively expensive.^9^

One of the most effective strategies to model molecular systems in complex environments has been the development of multiscale approaches.^10–15^ These methods focus on a part of the system, i.e., the target molecule, aiming to accurately capture its interactions with the environment while avoiding the computational cost of explicitly simulating the environment’s intrinsic properties. The fundamental assumption underlying these approaches is that the molecule’s energy and response properties are primarily local, influenced by—but not entirely dependent on—the surrounding environment.

In recent years, substantial progress has been made in developing multiscale quantum mechanical/molecular mechanical (QM/MM) methods, which provide an atomistic description of the entire system and allow detailed modeling of specific molecule-environment interactions.^16–18^ Many QM/MM approaches focus on describing electrostatic interactions between the QM and MM regions. Among these, the most accurate methods incorporate mutual polarization between the QM and MM subsystems.

This advancement has led to the development of several families of polarizable QM/MM approaches, including the Polarizable Embedding (PE) model of the Scandinavian school,^19^ QM/MMPol formulations from the Mennucci group,^20^ QM/AMOEBA schemes,^21,22^ induced-dipole models,^23–25^ Drude oscillators,^26^ fluctuating charges (FQ),^27–29^ and, more recently, fluctuating charges and dipoles (FQFμ).^18^ In the FQ­(Fμ) approach, the molecule–environment interaction is modeled by assigning each MM atom a charge (and, in the FQFμ case, also an induced dipole) that responds self-consistently to electronegativity differences between MM atoms and to the electrostatic potential generated by the QM density.

Various methods have already been coupled to FQ and FQFμ, enabling a more comprehensive description of molecular systems. For example, approaches such as Density Functional Theory (DFT) combined with Fluctuating Charges are well established,^18,29–31^ and more recent developments include Multiconfigurational Self-consistent Field (MCSCF) methods.^32^ In contrast, the coupling between Green’s function-based approaches and polarizable embeddings based on FQ and FQFμ has yet to be explored. The GW approximation, originally introduced by Hedin,^33^ is a Green’s function-based many-body perturbation theory method that provides an accurate description of electron correlation effects at moderate computational cost. Although initially developed and applied to periodic systems,^34–37^ GW has been successfully extended to small metal clusters^38^ and molecular systems,^39–48^ where it is now routinely employed to compute quasiparticle energies and spectral properties.^49–51^ Currently, several efforts have aimed to combine the GW formalism with quantum mechanical^52–56^ and classical environment models. Examples of the latter class of methods include the GW/COSMO approach^57–59^ recent implementations based on discrete polarizable embeddings, such as DRF,^60^ which is available within the AMS suite of programs employed in this work, and distributed atomic multipole models.^61,62^

In this article, we present the first formulation of the GW method combined with the FQ and FQFμ embedding approaches. This development enables the consistent inclusion of environmental polarization effects within the GW formalism and extends the applicability of polarizable QM/MM approaches to the accurate computation of charged excitations and quasiparticle properties.

## 2 Theory

### 2.1 Quantum/Classical Atomistic Embedding Approaches

The total energy (*E*_tot_) of a QM/MM system can be expressed as the sum of the quantum, classical, and interaction contributions:^31^

1 $$E_{\mathrm{tot}}=E_{\mathrm{QM}}+E_{\mathrm{MM}}+E_{\mathrm{int}}^{\mathrm{QM}/\mathrm{MM}}$$

where *E*_QM_ and *E*_MM_ are the energies of the QM and MM portions, respectively. By neglecting nonelectrostatic (dispersion/repulsion) interactions, the QM–MM interaction energy, $E_{\mathrm{int}}^{\mathrm{QM}/\mathrm{MM}}$ , can be expressed as

2 $$E_{\mathrm{int}}^{\mathrm{QM}/\mathrm{MM}}=E_{\mathrm{ele}}^{\mathrm{QM}/\mathrm{MM}}+E_{\mathrm{pol}}^{\mathrm{QM}/\mathrm{MM}}$$

where the electrostatic energy term $E_{\mathrm{ele}}^{\mathrm{QM}/\mathrm{MM}}$ and a possible polarization energy contribution $E_{\mathrm{pol}}^{\mathrm{QM}/\mathrm{MM}}$ are indicated. In a general force field representation, MM atoms can be characterized by a set of fixed multipole moments **M** (e.g., charges, dipoles, and quadrupoles) and a set of polarizable degrees of freedom **D**, which respond to the electrostatic potential and field generated by the QM region.

Depending on the definitions of **M** and **D**, and whether polarization effects are included, different embedding models can be formulated. Nonpolarizable schemes involve only **M**, while polarizable models additionally incorporate **D**.

Assuming a classical electrostatic interaction between the QM density and the MM embedding sites, the total energy in eq 1 can be rewritten as

3 Etot[ρ,D]=EQM[ρ(r)]+M†∫TM(r)ρ(r)dr+12D†AD+D†∫TD(r)ρ(r)dr+D†TM

where ρ­(**r**) is the QM density, the matrix **A** describes the self-interaction of the polarization sources, while **T** encodes the coupling between fixed (**M**) and polarizable (**D**) classical sites. The kernels **T**_ξ_(**r**)­(ξ = **M**, **D**) define the electrostatic interaction between the QM density and the MM distributions.^63–65^

Within the Kohn–Sham (KS) DFT framework, the effective QM/MM Fock matrix *F̃* is obtained by functional differentiation of eq 3 with respect to the electronic density ρ. Conversely, the stationarity of the total energy with respect to the classical polarization variables **D** yields the equations governing the polarization response of the MM region. This variational principle leads to a set of coupled QM/MM equations, in which the QM and MM subsystems interact through the mutual exchange of electrostatic and polarization fields. This allows us to define the coupled QM/MM equations as follows:

4 $$\frac{\delta E_{\mathrm{tot}}\left[\rho ,D\right]}{\delta \rho \left(r\right)}=h_{\mathrm{KS}}^{0}\left[\rho \left(r\right)\right]+{\mathrm{v̂}}_{\mathrm{emb}}\left(r\right)=\mathrm{F̃}$$

5 $$\frac{\delta E_{\mathrm{tot}}\left[\rho ,D\right]}{\delta D}=\Theta \left[\rho ,D\right]=0$$

where $h_{\mathrm{KS}}^{0}$ is the usual KS operator, given by

6 $$h_{\mathrm{KS}}^{0}=-\frac{1}{2}\nabla^{2}-\underset{m}{\sum }\frac{Z_{m}}{\left|r-R_{m}\right|}+\int \frac{\rho \left(r^{\prime }\right)}{\left|r-r^{\prime }\right|}dr^{\prime }+\frac{\delta E_{\mathrm{XC}}}{\delta \rho \left(r\right)}$$

where *E*_XC_ is the exchange–correlation energy functional. In eqs 4 and 5, *v̂*_emb_(**r**) and Θ­[ρ,**D**] are defined as

7 $${\mathrm{v̂}}_{\mathrm{emb}}\left(r\right)≔M^{†}T_{M}\left(r\right)+D^{†}T_{D}\left(r\right)$$

8 $$\Theta \left[\rho ,D\right]≔AD+\int T_{D}\left(r\right)\rho \left(r\right)dr+TM$$

The solutions to eqs 4 and 5 define the ground-state (GS) QM density and the polarization vector **D**. The equations presented constitute a general framework that can be adapted to the various embedding schemes considered in this work. These schemes differ in the specific definitions of **M** and **D** (see Figure 1):

**Figure 1:**
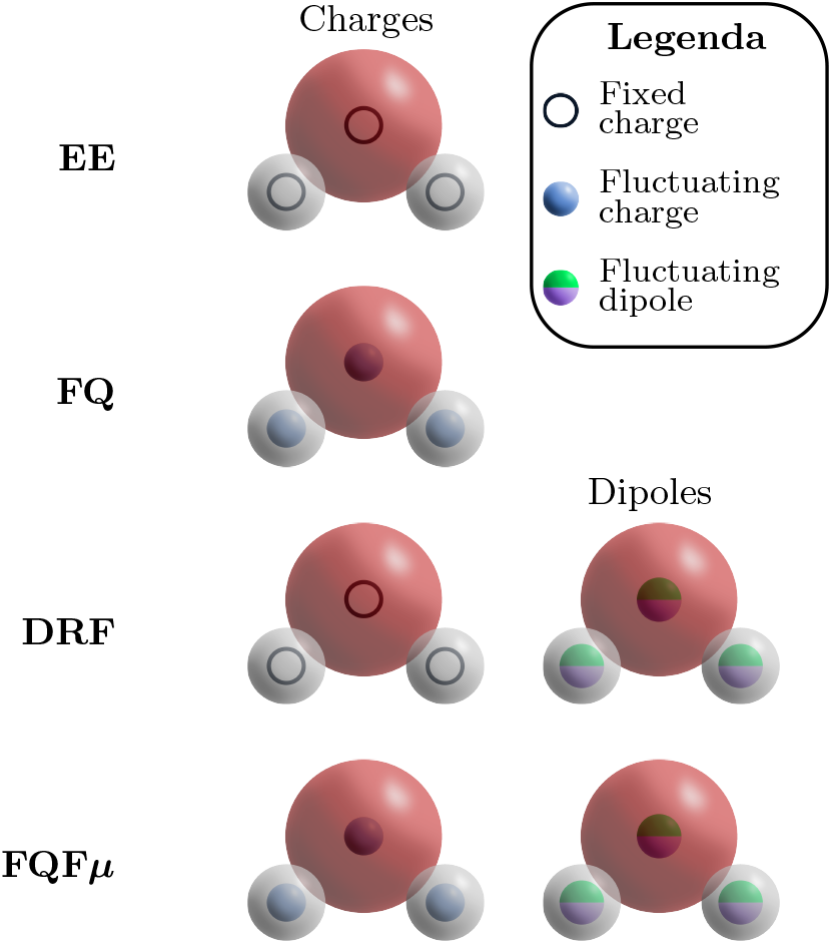
Schematic representation of QM/MM embedding models using a water molecule as a prototype.

1 Electrostatic Embedding (EE): Each atom in the MM region is endowed with a fixed charge, i.e., M = [qM] and D = [0]. Therefore, the MM layer polarizes the QM density but not vice versa; thus, it only indirectly affects the QM solute’s response properties.
2 Fluctuating Charges (FQ): Each MM atom is endowed with a charge whose value is not fixed but varies as a result of polarization effects.^29,31,66,67^ Thus, M = [0] and D = [q], with q corresponding to the polarizable charges. The parameters entering the FQ models, and thus determining the value of q, are the atomic electronegativity χ and chemical hardness η, which are theoretically defined in conceptual DFT.^68^ The polarization follows from the electronegativity equalization principle,^69,70^ which allows defining atomic partial charges in terms of the constrained minimum of a suitable energy functional.^31^
3 Discrete Reaction Field (DRF): In the DRF model, each MM atom carries a fixed permanent charge qM and a polarizable dipole μ that responds linearly to the electrostatic field generated by the QM density and by the other MM sites.^71–73^ In the notation introduced above, this corresponds to M = [qM] and D = [μ]. We emphasize that this is a classical, mean-field polarizable embedding scheme (often referred to as a discrete reaction field) and should not be confused with the Direct Reaction Field Hamiltonian, which introduces a genuine quantum-mechanical reaction-field operator acting on the electronic wave function.^74–77^

4 Fluctuating Charges and Fluctuating Dipoles (FQFμ): Each MM atom is endowed both with a polarizable charge q and a polarizable dipole μ.^18,78–81^ FQFμ is a pragmatic extension of the FQ model, where D = [q, μ]. The parameters that need to be set are the atomic electronegativity χ, chemical hardness η, and atomic polarizability α.

### 2.2 The GW Approximation within Density Functional Theory

The KS formulation of DFT represents the standard reference framework for Green’s function-based many-body perturbation theory (MBPT) calculations in molecular systems.^49,50^ Within this formalism, the time-ordered noninteracting KS Green’s function *G*_0_, expressed in the frequency domain, is defined as

9 $$G_{0}\left(r,r^{\prime },\omega \right)=\underset{n}{\sum }\frac{\phi_{n}\left(r\right)\phi_{n}^{*}\left(r^{\prime }\right)}{\omega -\varepsilon_{n}-i\eta \cdot \mathrm{sgn}\left(E_{F}-\varepsilon_{n}\right)}$$

where ϕ_*n*_(***r***) are the KS single-particle states and ε_*n*_ the KS eigenvalues. In this representation, *E*_*F*_ is the Fermi energy, and η is a positive infinitesimal, ensuring the correct analytic structure of the propagator. The full interacting Green’s function *G* is obtained by solving the Dyson equation

10 G(r,r′,ω)=G0(r,r′,ω)+∫G0(r,r1,ω)×Σ(r1,r2,ω)G(r2,r′,ω)dr1dr2

where Σ denotes the electron self-energy operator. The Dyson equation leads to a quasiparticle eigenvalue problem of the form:^82,83^

11 $${ĥ}_{0}\phi_{n}\left(r\right)+\int \Sigma^{\mathrm{XC}}\left(r,r^{\prime };E_{n}\right)\phi_{n}\left(r^{\prime }\right)dr^{\prime }=E_{n}\phi_{n}\left(r\right)$$

where *ĥ*_0_ is the mean-field Hamiltonian including kinetic energy, external potential, and Hartree terms. The solutions *E*_*n*_ of this equation are the poles of *G* and correspond to the exact electronic energies, which are accessible experimentally through (inverse) photoemission spectroscopy.^50^ The exchange-correlation contribution to the self-energy, Σ^XC^, is nonlocal and frequency-dependent, in contrast with the static potential *v*_*xc*_ typically employed in KS-DFT.^84^ In the GW approximation, it can be expressed in the frequency domain as the difference between the full self-energy Σ and the Hartree-exchange-correlation potential of KS-DFT, yielding:^57^

12 $$\begin{array}{ll} \Sigma^{\mathrm{XC}}\left(r,r^{\prime };E\right) & =\frac{i}{2\pi }\int d\omega e^{i\omega \eta }G\left(r,r^{\prime };E+\omega \right)W\left(r,r^{\prime };\omega \right)\\ & -v_{xc}\left(r,r^{\prime }\right) \end{array}$$

where *W* denotes the dynamically screened Coulomb interaction. It is constructed by solving the Dyson equation:

13 $$\begin{array}{ll} W\left(r,r^{\prime };\omega \right) & =v\left(r,r^{\prime }\right)+\int dr_{1}dr_{2}v\left(r,r_{1}\right)\\ & \times \chi_{0}\left(r_{1},r_{2};\omega \right)W\left(r_{2},r^{\prime };\omega \right) \end{array}$$

where *v*(***r***, ***r** ^**′**^*) is the bare Coulomb operator and χ_0_ is the independent-particle polarizability, defined as

14 $$\chi_{0}\left(r,r^{\prime };\omega \right)=\underset{i,j}{\sum }\left(f_{i}-f_{j}\right)\frac{\phi_{i}^{*}\left(r\right)\phi_{j}\left(r\right)\phi_{j}^{*}\left(r^{\prime }\right)\phi_{i}\left(r^{\prime }\right)}{\varepsilon_{i}-\varepsilon_{j}-\omega -i\eta \mathrm{sgn}\left(\varepsilon_{i}-\varepsilon_{j}\right)}$$

with *f*_*i*_ and *f*_*j*_ denoting orbital occupation numbers.

Eqs 9 [Disp-formula eq14] define the theoretical foundation of the GW approximation as applied to molecular systems and serve as the starting point for the incorporation of environmental effects via polarizable embedding models. The equations can be solved in a variety of ways, the most popular ones being by analytical integration^41,85,86^ on the imaginary frequency axis^87^ or combining imaginary frequency and imaginary time representations^88–93^ followed by analytical continuation (AC) to real frequencies^94,95^ contour deformation^96–98^ or merging contour deformation with AC.^99–101^ Covering all these techniques comprehensively is out of the scope of this work, and we instead refer to ref. 50 for an excellent review.

As in most works, here also we adopt the *G*_0_ *W*_0_ approximation in which the off-diagonal elements in eq 11 are neglected, and the self-energy is constructed from the KS Green’s function without updating the eigenvalues.^37,50^ In this case, eq 11 becomes

$$\epsilon_{n}+{[\Sigma_{xc}\left(E_{n}\right)-v_{xc}]}_{nn}=E_{n}$$

for each orbital indexed by *n*. The equation is nonlinear in *E*_*n*_ and might therefore have multiple solutions for different states *n*. In this case, one identifies the solution *E*_*n*_ for which the self-energy has the smallest slope as the so-called quasiparticle (QP) energy, while the other ones correspond to satellites. The QP energy equals the exact electron addition/removal energy of the system.

### 2.3 Coupling *G*_0_ *W*_0_ with Polarizable Embedding Models

Accurate evaluation of quasiparticle energies within the *G*_0_ *W*_0_ approximation, particularly in the presence of a molecular environment, requires the explicit inclusion of the environment’s polarizable response. To this end, we consider a general class of polarizable embedding models in which the classical region is described by atom-centered sources endowed with linear response degrees of freedom—specifically, polarizable monopoles (charges) and dipoles—whose values are determined by the electrostatic potential and electric field generated by the quantum density. This framework generalizes a wide range of QM/MM embedding schemes, including fluctuating charges (FQ), discrete reaction fields (DRF), and fluctuating charges and dipoles (FQFμ) as specific cases. In the limiting case where polarization effects are neglected and all classical degrees of freedom are fixed, the electrostatic embedding (EE) model is recovered.

Within this framework, the influence of the classical environment on the GW self-energy operator manifests in two distinct contributions. First, the so-called *implicit* contribution arises from the modification of the ground-state KS electronic structure due to the embedding potential. This effect is captured by performing the initial DFT calculation in the presence of the embedding field, thereby ensuring that the KS eigenvalues and orbitals reflect the polarized environment. Second, and more critically within the GW formalism, an *explicit* contribution arises from the dynamic response of the polarizable environment, which modifies the screening of the Coulomb interaction (see Figure 2). This environmental screening must be incorporated in the definition of the screened Coulomb operator *W*_0_ and is closely related to the local-field effects discussed in continuum and discrete embedding models.^15,57,58,102–104^

**Figure 2:**
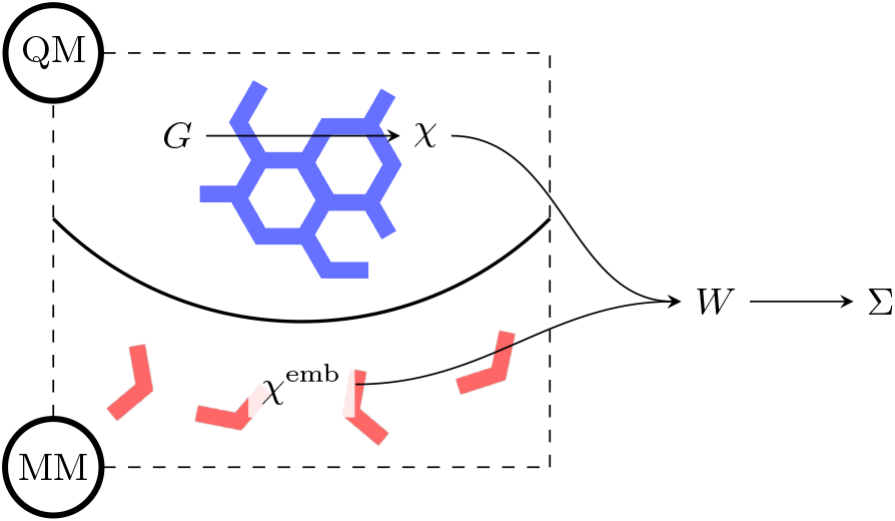
Schematic representation of the *G*_0_ *W*_0_/FQ­(Fμ) approach. The total polarizability includes both quantum (χ) and classical (χ^emb^) contributions, which in turn affect the screening described by *W*_0_. The screening is then used to evaluate the self-energy (Σ).

To formalize the derivation, we first recall the quantities introduced above and define the additional notation used in this section:

-
dQ denotes an infinitesimal test charge placed at position **r**′ within the quantum region. Its contribution to the electronic density can be written as $dQ\delta \left(r-r^{\prime }\right)$ , with δ the Dirac delta distribution.
- **D** is the vector collecting all classical polarizable multipoles.
- d**D** represents the variation of **D** induced by the presence of the infinitesimal test charge dQ .

At the ground-state stationary point, eq 5 together with the definition of Θ in eq 8 gives

15 $$0=AD+\int T_{D}\left(r\right)\rho \left(r\right)dr+TM$$

Introducing a perturbation of the quantum density in the form of the infinitesimal test charge dQ leads to the linearized condition

16 $$0=AdD+\int T_{D}\left(r\right)dQ\delta \left(r-r^{\prime }\right)dr$$

which provides the response of the classical polarization degrees of freedom to the perturbation.

So that the response of the classical subsystem is governed by the linear system

17 $$-AdD=T_{D}\left(r^{\prime }\right)dQ$$

Here, **A** is the response matrix of the embedding model, incorporating both self-and mutual interactions among the classical polarizable sites, while **T**_**D**_(**r**′) encodes the coupling between the quantum perturbation and the classical polarization degrees of freedom.

From eq 17 it is clear that the variation of the classical polarization depends solely on quantities associated with the polarizable part of the environment, namely, the matrix **A** and the polarization variables collected in **D**. In particular, fixed electrostatic multipoles do not contribute to this response, which is entirely driven by the polarizable degrees of freedom.

Once d**D** is known, the reaction field potential induced at a second quantum point **r** is obtained via:

18 $$dv^{\mathrm{res}}\left(r,r^{\prime }\right)=dD^{†}T_{D}\left(r\right)$$

As shown in different contexts,^54,105^ the reaction potential enters in the effective Coulomb interaction as

19 $$ṽ\left(r,r^{\prime }\right)=v\left(r,r^{\prime }\right)+dv^{\mathrm{res}}\left(r,r^{\prime }\right)$$

which is used to construct the fully screened Coulomb potential in the frequency domain:

W0(r,r′;ω)=ṽ(r,r′)+∬dr1dr2ṽ(r,r1)×χ0(r1,r2;ω)W0(r2,r′;ω) 20

where χ_0_ is the independent-particle susceptibility of the quantum subsystem.

This general formulation is consistent with recent GW/MM approaches developed for continuum^57^ and discrete polarizable models,^58,64^ ensuring that only the physically meaningful, dynamically responsive components of the environment—those included in **D**—contribute explicitly to the screening operator *W*_0_. Here fixed electrostatic sources **M** enter only the ground-state potential and influence the quasiparticle energies indirectly.

To quantify the impact of the environment on the electron–electron interaction, we evaluate the reaction potential induced by the classical region in response to an infinitesimal perturbation in the quantum subsystem.

In the case of a charge-based model, the response of the classical environment to a quantum perturbation as seen in eq 17 can be expressed through the evaluation of the electrostatic potential of the classical (MM) region:

21 $$-AdD=k_{C}T^{\left(0\right)}\left(r^{\prime }\right)dQ$$

where we have used the fact that, for charge–charge interactions, the coupling between the quantum perturbation dQ and the classical embedding sites in positions ***r***_*t*_ reduces to the Coulomb kernel multiplied by the Coulomb constant, namely

22 $$T_{t}^{\left(0\right)}\left(r^{\prime }\right)=\frac{1}{\left|r^{\prime }-r_{t}\right|}$$

23 $$k_{C}=0.7853\;\mathrm{a.u.}$$

Within the FQ model, the classical region responds linearly to these perturbations. The variation in the atomic classical charges d ***q*** of the classical portion can be obtained by solving the following linear system:

24 $$\left(\begin{array}{cc} T^{\mathrm{qq}} & 1_{\lambda }\\ 1_{\lambda }^{†} & 0 \end{array}\right)\left(\begin{array}{c} dq\\ 0 \end{array}\right)=\left(\begin{array}{c} -k_{C}T^{\left(0\right)}\left(r^{\prime }\right)\\ 0 \end{array}\right)dQ$$

where we recognize the structure of eq 21. Let us denote the inverse of the system matrix by the blockwise structure:

25 $${\left(\begin{array}{cc} T^{\mathrm{qq}} & 1_{\lambda }\\ 1_{\lambda }^{†} & 0 \end{array}\right)}^{-1}=\left(\begin{array}{cc} B^{\mathrm{qq}} & b_{1}\\ b_{2} & b_{3} \end{array}\right)$$

The variations of the classical polarization sources are then given by

26 $$dq=-k_{C}B^{\mathrm{qq}}T^{\left(0\right)}\left(r^{\prime }\right)dQ$$

The reaction potential acting back on the quantum system is then expressed as

27 $$dv^{\mathrm{res}}\left(r\right)=k_{C}T^{(0)†}\left(r\right)dq$$

By substituting the expressions from eq 26, we obtain

28 $$dv^{\mathrm{res}}\left(r\right)=-{k_{C}}^{2}T^{(0)†}\left(r\right)B^{\mathrm{qq}}T^{\left(0\right)}\left(r^{\prime }\right)dQ$$

This reaction potential effectively modifies the bare Coulomb interaction within the quantum subsystem. In the context of the GW formalism, this contribution must be incorporated into the definition of the screened interaction *W*_0_ as defined in eqs 19 and 20.

It is worth noticing that the structure of eq 21 closely resembles the polarization term appearing in the Hamiltonian of the Direct Reaction Field approach.^74,75,106,107^ This method should not be confused with the DRF (Discrete Reaction Field) model discussed above, which treats the environment as a classical polarizable medium responding to the mean electrostatic field of the QM density. In the Direct Reaction Field formalism, the polarization of the environment is instead expressed directly in terms of the quantum mechanical density operator, so that the reaction field enters the electronic Hamiltonian as an explicit one- and two-electron operator rather than through a purely mean-field embedding potential.^76,77^ This construction enables a treatment of environmental polarization that goes beyond the usual mean-field description and can be consistently combined with correlated wave function methods.

To further enhance the description of polarization effects in classical embedding models, it is possible to include not only polarizable charges but also polarizable dipoles. This approach is adopted in models such as FQFμ and DRF, and it provides a more general framework applicable to a broader class of polarizable force fields.

In the case of a system characterized by both polarizable charges and polarizable dipoles, it is necessary to also account for the contribution of the electric field induced by the test charge introduced in eq 17. In addition to the variation of the electrostatic potential d­[*v* ^ext^], as defined in eq 21, we must compute the α-component (α = *x*, *y*, *z*) of the electric field $d\left[E_{\alpha }^{\mathrm{ext}}\right]$ generated by the infinitesimal test charge d[Q] placed at position **r**_*t*_:

29 $$dE_{\alpha }^{\mathrm{ext}}\left(r_{t}\right)=k_{C}T_{\alpha ,t}^{\left(1\right)}\left(r^{\prime }\right)dQ$$

where *k*_C_ = 0.7853 a.u. is the Coulomb constant, and $T_{\alpha ,t}^{\left(1\right)}\left(r^{\prime }\right)$ is the electrostatic kernel for a dipole:

30 $$T_{\alpha ,t}^{\left(1\right)}\left(r^{\prime }\right)=\frac{{r^{\prime }}_{1,\alpha }-r_{t,\alpha }}{{|r_{1}^{\prime }-r_{t}|}^{3}}$$

To generalize the model and account for additional sources of polarization, we now introduce atom-centered induced dipoles in addition to fluctuating charges. Assuming a linear response of the classical region to external perturbations, the coupled response of charges and dipoles can be obtained by solving the following linear system:

31 $$\left(\begin{array}{ccc} T^{\mathrm{qq}} & 1_{\lambda } & T^{q\mu }\\ 1_{\lambda }^{†} & 0 & 0\\ {T^{q\mu }}^{†} & 0 & T^{\mu \mu } \end{array}\right)\left(\begin{array}{c} dq\\ 0\\ d\mu \end{array}\right)=\left(\begin{array}{c} -k_{C}T^{\left(0\right)}\left(r^{\prime }\right)\\ 0\\ k_{C}{T_{\alpha }}^{\left(1\right)}\left(r^{\prime }\right) \end{array}\right)dQ$$

where the inverse of the matrix on the left-hand side can be written in block form as

32 $${\left(\begin{array}{ccc} T^{\mathrm{qq}} & 1_{\lambda } & T^{q\mu }\\ 1_{\lambda }^{†} & 0 & 0\\ {T^{q\mu }}^{†} & 0 & T^{\mu \mu } \end{array}\right)}^{-1}=\left(\begin{array}{ccc} B^{\mathrm{qq}} & b_{1} & B^{q\mu }\\ b_{2} & b_{3} & b_{4}\\ B^{\mu q} & b_{5} & B^{\mu \mu } \end{array}\right)$$

The infinitesimal variation of the MM charges and dipoles induced by the perturbation can thus be expressed as

33 $$\left\{ \begin{array}{l} dq=k_{C}\left(-B^{\mathrm{qq}}T^{\left(0\right)}\left(r^{\prime }\right)+B^{q\mu }T^{\left(1\right)}\left(r^{\prime }\right)\right)dQ\\ d\mu =k_{C}\left(-B^{\mu q}T^{\left(0\right)}\left(r^{\prime }\right)+B^{\mu \mu }T^{\left(1\right)}\left(r^{\prime }\right)\right)dQ \end{array}\right.$$

It is important to highlight that, contrary to the case of fluctuating charges alone (see eq 26), the variation of the MM charges here includes a contribution from the dipolar kernel via the cross term **B** ^**qμ**^ ***T*** ^(1)^.

Finally, the total response potential acting on the quantum region can be obtained similarly to eqs 27 and 28, and is given by

dvres(r)=kC2T(0)†(r)(−BqqT(0)(r′)+BqμT(1)(r′))dQ+kC2T(1)†(r)(−BμqT(0)(r′)+BμμT(1)(r′))dQ 34

This expression accounts for the contributions of both induced charges and dipoles, allowing for a more accurate description of polarization effects in polarizable embedding models.

Finally, we emphasize that the derived formalism is general and can be straightforwardly extended to other polarizable embedding models, including discrete reaction field (DRF)^71–73^ and implicit Conductor-like Screening Model (COSMO) approaches,^108–110^ provided they rely on charge and/or dipole-based polarization responses. In fact, DRF in its basic form uses static charges (which do not contribute directly to the polarizability terms that go into the GW equation) and polarizable dipoles that can be treated in the exact same way as the fluctuating dipoles of FQFμ, with the only difference being the form of the matrix that yields the electric field from the dipoles themselves, which depends on the parametrized atomic polarizabilities.

### 2.4 Implementation

At the time of its development, one of the key goals of the ADF implementation was to overcome the computational bottleneck associated with the $O\left(N^{4}\right)$ scaling of electron repulsion integral (ERI) evaluation and manipulation.^60^ To address this, the calculation of the Coulomb potential was reformulated by using an auxiliary basis set (ABS) composed of atom-centered Slater-type orbitals (STOs), allowing for an efficient expansion of the electron density.

In ADF, matrix elements are computed entirely through a three-dimensional numerical integration. The electrostatic potentials at each grid point are evaluated using the auxiliary density representation, which enables accurate and efficient computations.^111^

Analogous to the Coulomb potential, the electrostatic response potential *v* ^res^ for a given subsystem *I* must also be evaluated on the basis of auxiliary fitting functions. Specifically:

35 $$\begin{array}{ll} v_{ij} & =\iint f_{i}\left(r\right)\frac{1}{\left|r-r^{\prime }\right|}f_{j}\left(r^{\prime }\right)drdr^{\prime }\\ dv_{ij}^{\mathrm{res}} & =\iint f_{i}\left(r\right)v^{\mathrm{reac}}\left(r_{I},r_{I}^{\prime }\right)f_{j}\left(r^{\prime }\right)drdr^{\prime } \end{array}$$

The specific form of *v* ^reac^ depends on the polarizable embedding model adopted. Different models lead to distinct expressions for the reaction potential, reflecting their underlying treatment of the environment’s response. The expressions of *v* ^reac^ for FQ and FQ­(Fμ) have been given in Section 2.3.

As soon as δ*v* ^res^ and χ_0_ are available in the ABS, we solve eq 20 to obtain *W* in the ABS, which is then used to evaluate the self-energy eq 12, which only requires trivial modifications of existing GW implementation.^57^ In this way, the response to polarizable environments can be included in the cubic-scaling GW implementations^89–92^ which follow the space-time method,^112,113^ as well as implementations that work on the imaginary frequency axis only and evaluate χ_0_ in $O\left(N^{4}\right)$ .^87^ We have implemented both variants, but all results reported here have been obtained working on the imaginary frequency axis only, followed by AC using a Padé approximant.^94^

## 3 Computational Details

The computational strategy adopted in this work builds upon previously established procedures developed by some of us.^31,114^ We take as an example a molecular system (4-hydroxybenzylidene-1,2-dimethylimidazolinone, p-HDBI; see Figure 4) embedded in aqueous solution, and we employ a fully atomistic description of the solute–solvent system and a multistep protocol, schematically illustrated in Figure 3.

**Figure 3:**
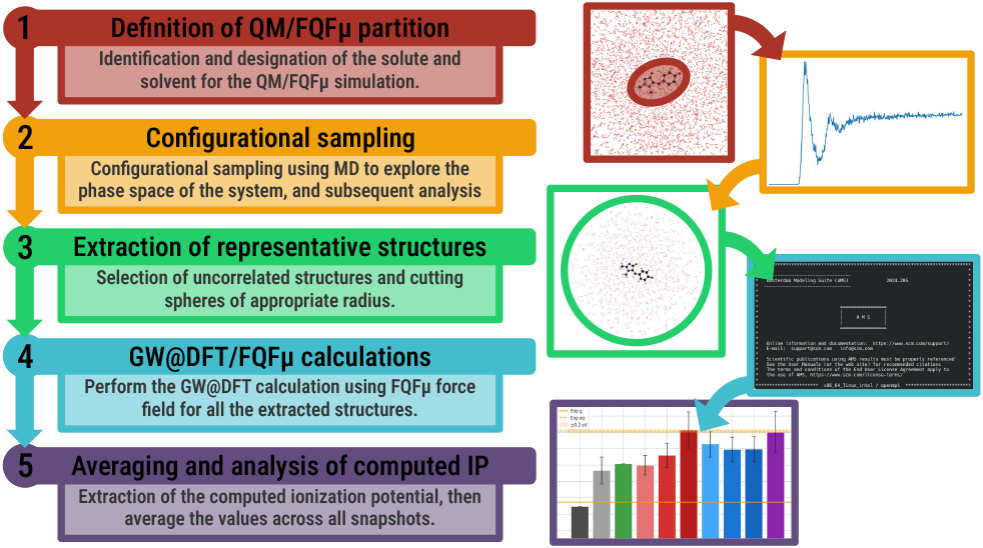
Schematic representation of the multistep computational protocol employed in this work. The protocol consists of five steps: (1) definition of the QM/MM partition; (2) configurational sampling; (3) extraction of a set of representative conformations; (4) GW@DFT/MM calculations; and (5) analysis of computed energies.

p-HDBI is the chromophore that lies at the heart of Green Fluorescent Protein (GFP) and is anchored covalently and by a network of hydrogen bonds to the protein that is wrapped around it in a β-barrel structure.^5^

To ensure an accurate description of the solvation effects and electronic response, we employed classical molecular dynamics (MD) simulations to generate ensembles of uncorrelated solvent configurations around a fixed solute geometry. For p-HBDI, two alternative restraint schemes were applied to enforce the aromatic rigidity, differing in the degree of intramolecular flexibility that was allowed. Further computational details are provided in the (Supporting Information SI). A representative subset of these snapshots is selected and used to perform *G*_0_ *W*_0_/MM calculations. The MM region includes all solvent molecules within the cutoff radius and is treated by using either fixed-charge or polarizable force fields, depending on the model considered.

*G*_0_ *W*_0_ calculations are performed using the settings recommended for accurate gas-phase results,^49,115^ with molecular orbitals represented in a basis of Slater-type orbitals (STOs).^115,116^ In particular, we adopt the Corr/TZ3P and Corr/QZ6P basis sets,^115^ which have been shown to offer a good balance between accuracy and computational cost. Converging individual QP energies to the complete basis set (CBS) limit is rarely possible^117,118^ and therefore an extrapolation is often performed.^49,85,119,120^ We here follow previous work and extrapolate to the CBS limit using the correlation-consistent basis sets Corr/TZ3P and Corr/QZ6P.^115,121^ The extrapolation follows the standard two-point formula, as described in the AMS documentation.^60^ In all calculations, we used 25 G-Legendre grid points^122^ to sample the imaginary frequency axis.

Regarding the choice of exchange–correlation functional for the KS reference, a preliminary benchmark was conducted on the phenol molecule (Figure 4). The functional that yielded the most consistent results with the available reference data was subsequently selected for production calculations.

**Figure 4:**
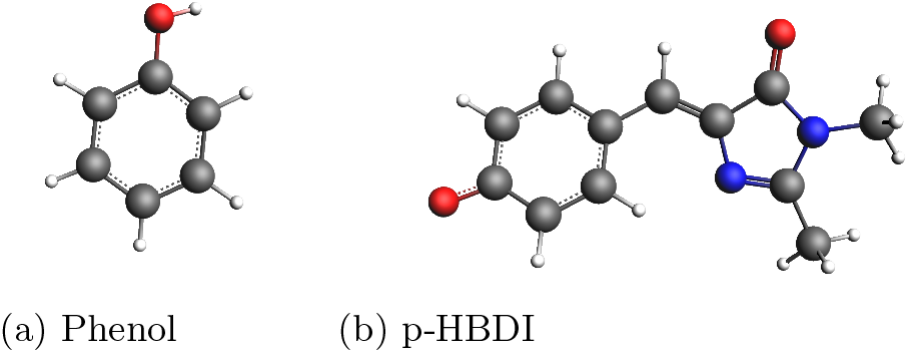
Molecular structures of the systems studied in this work: (a) is phenol (b) is p-HBDI.

The MM portion is described using four different atomistic schemes: a nonpolarizable force field, FQ,^27^ DRF,^71,72^ and FQFμ.^18^ In particular, we adopt three different parametrizations for the FQ: FQ1 from ref. 27, FQ2 from ref. 123, and FQ3 from ref. 124, while for FQFμ the parameters are taken from ref. 18.

As a reference, we also perform *G*_0_ *W*_0_ calculations in the gas phase and by using the implicit continuum COSMO^108–110^ solvation approach.

MD simulations are performed using GROMACS,^125–130^ while all *G*_0_ *W*_0_ (classical) calculations are carried out using a locally modified development version of AMS.^60^

Concerning the computational cost of our method, the additional overhead associated with the embedding is moderate. As a rule of thumb, a single-shot *G*_0_ *W*_0_ calculation typically requires a wall time comparable to that of a self-consistent calculation with a hybrid exchange–correlation functional; consequently, an SCF calculation followed by a *G*_0_ *W*_0_ step is roughly twice as expensive as a standalone hybrid-DFT calculation.^131^ This behavior is well established for gas-phase calculations and is essentially preserved in the embedded case. In particular, the routines introduced to account for the classical environment are computationally inexpensive compared to the GW part so that the overall timing of the calculation is not significantly affected by the presence of the embedding.

### 3.1 Calculation of the Ionization Potential

Before presenting the numerical results, we briefly outline the computational strategy adopted for evaluating the ionization potential (IP) within the *G*_0_ *W*_0_ framework combined with polarizable embedding models. The ionization potential, also termed ionization energy, represents the minimum energy required to remove an electron from a molecule in the gas phase and is a key quantity for characterizing electronic structure and reactivity.

The first ionization potential corresponds to the process:

36 $$X_{g}\to X_{g}^{+}+e^{-}$$

and defines the energy difference between the neutral and cationic ground states.

Experimentally, IPs are often determined via photoelectron spectroscopy, in which electrons are ejected by UV or X-ray radiation.^5,132–134^ The measured kinetic energy of the emitted electrons provides direct access to the energy levels of the system, including HOMO, from which the IP is obtained.

In the present work, we compute the ionization potential by evaluating the highest occupied quasiparticle energy obtained from MBPT. Within the *G*_0_ *W*_0_ approximation, the IP is defined as

37 $$\mathrm{IP}=-\varepsilon_{\mathrm{HOMO}}^{GW}$$

where $\varepsilon_{\mathrm{HOMO}}^{GW}$ is the quasiparticle energy associated with the HOMO level. The GW QP energies take into account the dynamic screening effects of the electron–electron interaction. As such, the GW-derived IPs provide a physically rigorous and quantitatively accurate description of charged excitations, particularly in the context of the present work, when the surrounding environment is properly treated through polarizable embedding.

## 4 Numerical Results

The first part of this section focuses on aqueous phenol. This small neutral molecule is employed to evaluate the performance of our novel methodology with respect to key computational parameters. In particular, we assess the impact of the starting point for the GW calculation—namely, the underlying DFT wave function—and the choice of MM force field used to describe the atomistic environment.

In the second part, we extend our analysis to p-HBDI, the chromophore of the GFP. This molecule is of particular interest not only due to its biological significance but also because it carries a negative charge, making the accurate description of solute–solvent interactions and polarization effects especially critical.

### 4.1 Phenol in Aqueous Solution

In this section, we focus on the study of phenol in an aqueous solution. This compound is a highly water-soluble species that has recently been investigated through high-resolution photoelectron spectroscopy.^134^ That study has revealed that the IP of phenol is strongly dependent on the nature of the solvent, underscoring the importance of accurately modeling the solvation effects.

Here, we exploit phenol as a benchmark system to test and validate the performance of our computational protocol. First, we assess the dependence of the computed IP on the choice of the starting point for the *G*_0_ *W*_0_ calculation, namely, the exchange–correlation functional employed in the underlying DFT calculation. Next, we compare results obtained at the KS and *G*_0_ *W*_0_ levels, while varying the embedding approach. Finally, we juxtapose the results obtained in the gas phase and in solution using different solvation models to identify the approach that best reproduces the experimental findings.

#### 4.1.1 Choice of the Functional

We evaluate the influence of the exchange–correlation functional on the calculated IP of phenol in aqueous solution. As shown in Figure 5, the starting point dependence is found to be rather limited in the present context. While the KS values vary significantly with the functional—as expected, due to differing amounts of exact exchange and self-interaction error correction—the corresponding *G*_0_ *W*_0_ ionization potentials are remarkably stable across all cases considered. This suggests that the GW correction efficiently compensates for the limitations of the KS reference, yielding consistent quasi-particle energies regardless of the functional used to generate the starting orbitals. This observation is particularly relevant in the context of solvated systems, where one might expect greater functional sensitivity due to the combined effects of electronic screening and solvent-induced polarization. Nevertheless, our results indicate that once the solvent environment is properly treated, the functional dependence of the GW IP becomes marginal. This validates the robustness of our computational setup and justifies the use of a single, well-performing functional for the remaining calculations.

**Figure 5:**
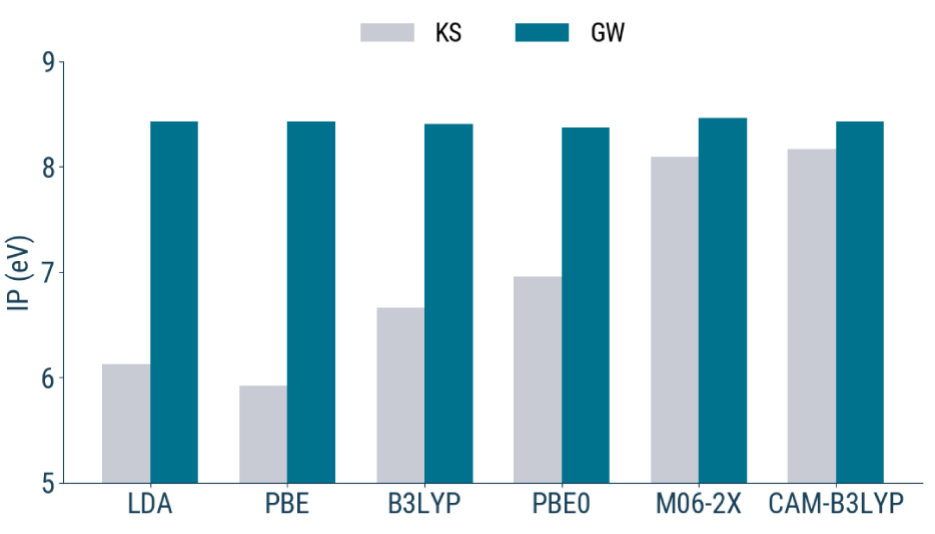
Comparison of KS-DFT (gray) and *G*_0_ *W*_0_ (blue) energies of the HOMO for phenol in aqueous solution using different functionals with the Corr/QZ6P basis set. Results are shown for a single representative snapshot.

Based on these findings, we selected the PBE0 functional as the starting point for all subsequent calculations. While functionals with higher amounts of exact exchange or range-separated hybrids typically provide better starting points for *G*_0_ *W*_0_ ^135,136^ calculations for isolated organic molecules, this choice is further motivated by its use in the literature in similar embedding approaches.^57,61^

#### 4.1.2 Comparison between Different Embedding Approaches

We analyze the effect of the *G*_0_ *W*_0_ correction relative to the values obtained from standard KS-DFT calculations, as plotted in Figure 6. Overall, we observe that the distribution of ionization potentials retains its shape across different embedding schemes, but it is shifted in energy depending on the force field employed.

**Figure 6:**
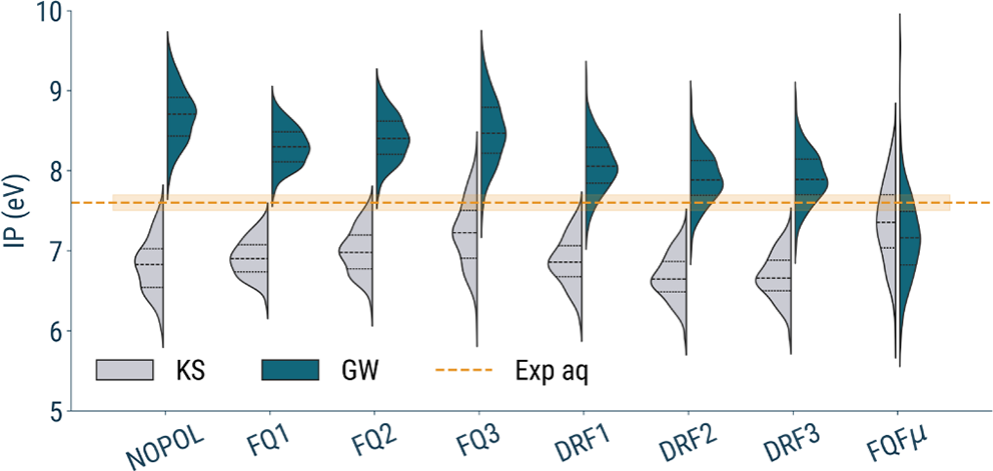
Violin plot of the HOMO energies computed on 200 uncorrelated snapshots of solvated phenol by using different embedding models. The gray distributions correspond to KS values, while the blue distributions refer to *G*_0_ *W*_0_ results. The experimental reference in the aqueous phase is indicated by the dashed line; the orange-shaded area represents the error bar of the experiment.

The first notable observation is that the nonpolarizable force field results in a distribution with the highest mean value (8.69 eV). This behavior can be attributed to the fact that, in the absence of polarizable embedding, no additional screening is introduced at the GW level from the classical environment. Consequently, the quasiparticle correction acts on a rigid electrostatic background, resulting in higher predicted ionization potentials. When comparing the FQ and DRF models, the GW-corrected IPs show similar overall shifts, around 1.2 eV. The differences between their mean values appear to be primarily driven by the KS starting point since the GW correction responds to the initial electronic structure defined at the DFT level.

A more pronounced shift is observed for the FQFμ model. This behavior can be rationalized by noting that, in contrast to FQ and DRF, whose response is governed by a single class of polarizable degrees of freedom (respectively charges or dipoles), the FQFμ Hamiltonian includes both fluctuating charges and fluctuating dipoles. As a consequence, the environmental response matrix contains not only the charge–charge or dipole–dipole blocks, but also the charge–dipole and dipole–charge coupling terms. All these contributions enter the reaction-field kernel that ultimately modifies the screened Coulomb interaction, thereby enhancing the overall effective polarizability of the embedding environment.

While this increased flexibility in the classical response may lead to a stronger screening and, consequently, to larger quasiparticle shifts, we emphasize that the present data do not allow us to determine whether this effect reflects a genuine physical enhancement of environmental polarization or simply the intrinsic overresponsiveness of the FQFμ parametrization employed here. A more systematic assessment would require either alternative parametrizations or dedicated reference data, which lie beyond the scope of the present study. We therefore limit ourselves to noting that the FQFμ formalism introduces additional polarization channels absent in FQ and DRF, and that these may account for the larger shifts observed.

Figure 7 presents computed averaged IP solvatochromic shifts for phenol in an aqueous solution. The calculations were carried out with both triple-ζ-correlated consistent and quadruple-ζ-correlated consistent basis sets, and the results were further extrapolated to the complete basis set (CBS) limit, as detailed in the Computational Details section of the SI.

**Figure 7:**
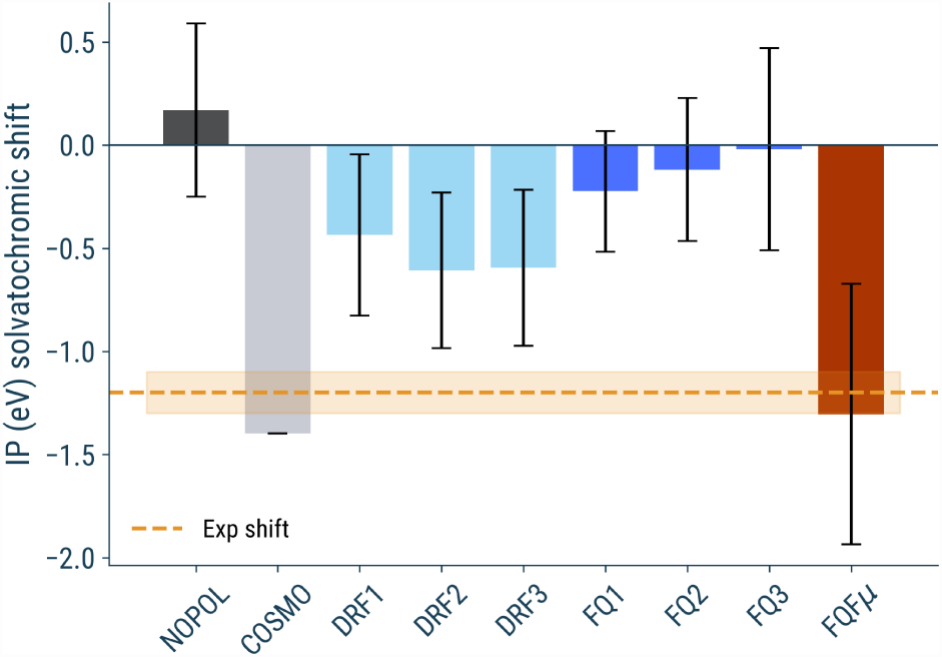
Shift of IP of phenol in aqueous solution with respect to vacuum computed on 200 uncorrelated snapshots. Values are obtained through extrapolation to a complete basis set based on Corr/TZ3P and Corr/QZ6P values. Bars indicate standard deviations. Horizontal orange line correspond to the experimental IP solvatochromic shift.^5^

First, we mention that the IP calculated in vacuum (8.55 eV) only slightly underestimates the experimental gas-phase reference value (8.8 eV),^134^ confirming the robustness of our protocol for isolated systems (all raw values are reported in Supporting Information). The slight underestimation of the first IP is the expected result for a *G*_0_ *W*_0_@PBE0 calculation of small to medium organic molecules.^137,138^ When moving to solvation approaches, significant differences emerge depending on the treatment of the environment. In particular, the nonpolarizable model (NOPOL, 0.17 eV) shows a solvatochromic shift in the wrong direction with respect to the experimental shift (around 1.2 eV), likely due to the absence of polarization effects in the classical embedding, which results in inadequate stabilization of the ionized state. On the other hand, the implicit solvation model (COSMO, −1.40 eV) slightly overestimates the experimental shift, probably reflecting a poor description of specific solute–solvent interactions (hydrogen bonding). Explicit polarizable models display more nuanced behavior depending on their parametrization and the specific way to include polarization effects, suggesting that polarization contributes to a more accurate representation of the solvation-induced screening effects in the *G*_0_ *W*_0_ calculations. FQ and DRF generally predict values between the gas-phase and aqueous experimental IPs, whereas FQFμ yields a value (1.3 eV) that is slightly below the experimental benchmark but still within one standard deviation.

### 4.2 p-HBDI in Aqueous Solution

In this section, we extend our analysis to p-HBDI, the chromophore of Green Fluorescent Protein (GFP). Unlike phenol, p-HBDI is negatively charged, making its electronic properties particularly sensitive to solvation effects. Therefore, accurate modeling of polarization and electrostatic interactions with the solvent is crucial. We report here the results obtained from geometries extracted from molecular dynamics simulations without applying strong positional restraints; further discussion is provided in the SI.

The calculated IPs for the p-HBDI molecule in aqueous solution, obtained from 200 uncorrelated snapshots, are shown in Figure 8. Starting from the vacuum result, our calculated value of 2.44 eV is in agreement with the experimental value of 2.73 eV reported in ref. 5. The experimental value for the solvated molecule, reported in,^5^ is significantly higher (around 7 eV) than the vacuum value, giving a solvatochromic shift of about 4.3 eV; this is expected, since solvation stabilizes the anionic electronic structure. The calculated IPs in aqueous solution are highly dependent on the force field employed. As expected, the nonpolarizable force field (3.8 eV) underestimates the experimental shift. This is likely because the nonpolarizable force field cannot account for the polarization of the solute’s negative charge by the solvent. COSMO predicts an even lower value (3.0 eV) compared to the nonpolarizable model, probably due to the lack of modeling any directionality in the solvation, therefore still not approaching the experimental value.

**Figure 8:**
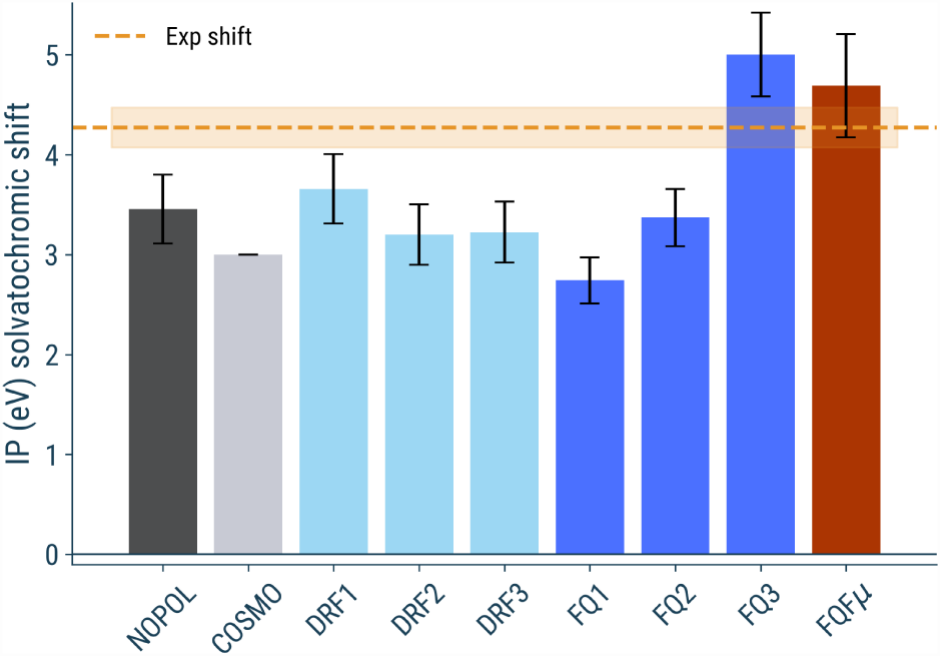
Averaged computed IPs shift for p-HBDI in aqueous solution with respect to vacuum by using various solvation models. The reported values represent the average of the HOMO quasiparticle energy at the *G*_0_ *W*_0_ level, and the associated error bars indicate the standard deviation of the distribution. Values based on implicit solvation models (COSMO) are also shown for reference. Horizontal orange line correspond to the literature reference shift value^5,139^ for the ionization potential of p-HBDI; the shaded region corresponds to an estimated uncertainty of ±0.2 eV. All raw values are reported in the (SI Table S2).

Moving to the FQ values, we observe that the effect of different parametrizations is crucial. While FQ1 (2.8 eV) significantly underestimates the IP, FQ3 (5.1 eV) provides a value that is closer to the experimental one. In the case of the DRF model, the parametrization has a smaller effect compared to the variability observed for FQ, with all shifts lying between 3.3 and 3.7 eV. Finally, the FQFμ model, which incorporates both charge and dipole polarization sources, provides an IP of 4.8 eV, nicely matching the experimental value considering the standard deviation of the distribution.

## 5 Conclusion and Future Perspectives

The focus of this work was primarily on the development of a multiscale QM/classical method, coupling the GW approximation (GWA) and the Fluctuating Charges and Fluctuating Dipoles (FQFμ) force field. This integration allows for modeling mutual polarization effects, where the quantum system and the classical environment influence each other’s electronic structures. This mutual influence is essential for accurately modeling systems where environmental effects, such as solvation, play a critical role.^31,114,140^ Selected case studies were used to validate the methodology, focusing primarily on the ionization potential (IP) calculations of embedded systems. The IP is a crucial property in quantum chemistry and is often employed to evaluate the performance of GW methods.

The first case study, phenol, explored how different functionals affect the calculated IP. It was observed that the GW method reduces the variability caused by these parameters, providing more stable and consistent results, with the PBE0 emerging as a good compromise. Also, different embedding techniques impact IP. The GW correction generally raises IP compared to KS, except in the case of FQFμ, where the increased polarization arising from charges and dipoles leads to a significant reduction in IP due to enhanced screening effects of the solvating environment.

In the second case study, the GFP chromophore, our calculations using GW/FQFμ and GW/FQ3 methods aligned closely with experimental data, reproducing the measured IP in aqueous solution. It has been shown that atomistic approaches, such as FQ1, FQ2, and DRF, tend to perform similarly in estimating the IP, while FQFμ predicts larger shifts. Overall, the GW results were in good agreement with the experimental data.

The *G*_0_ *W*_0_/FQ­(Fμ) framework can, in principle, be extended to more advanced formulations such as eigenvalue self-consistent GW (evGW),^43^ quasiparticle self-consistent GW (qsGW),^131,141–144^ and fully self-consistent GW (scGW),^145,146^ as discussed in Section 2.2. From a theoretical standpoint, the extension to *evGW* is straightforward and fully compatible with the present formalism, requiring only the iterative update of the quasiparticle energies. In contrast, implementations of qsGW and scGW pose significantly greater challenges due to the need for a fully self-consistent update of both energies and orbitals and would require a more profound restructuring of the electronic structure procedure within the embedding framework. Nevertheless, both extensions remain conceptually grounded in the formalism developed here, and recent studies^57–59^ suggest promising directions for their future realization.

Furthermore, the GW/FQ­(Fμ) formalism can be applied to calculate low-lying excitonic states of molecular systems through the Bethe-Salpeter equation (BSE).^59,61,147^ GW-BSE is a promising approach for calculating excited states due to its ability to model both local^148^ and charge transfer excitations^59,149^ while maintaining a favorable computational scaling.^84,150^ The approach is also easily extended to emerging Green’s function-based methods, which go beyond the GW approximation.^151–155^

In addition to refining the treatment of the QM portion of the system, the embedding approach can be improved to better capture short-range solute–solvent effects.^53,54^ Several strategies exist, one of which is a three-layer partitioning scheme, where the system is divided into three distinct regions. In this model, a minimal portion of the environment is described at the QM′ level (generally different from the QM level employed for the solute), while the remaining part is modeled classically. The addition of the intermediate QM′ layer permits us to effectively capture dispersion and repulsion interactions. In line with previous works on the development of DFT/FDE/FQ­(Fμ) approaches,^156,157^ GW methodologies can be coupled to FDE/FQFμ by taking inspiration from a recently proposed coupling of GW approaches within a quantum embedding formalism.^158^

## Supplementary Material



## Notes

Additional theoretical background on the GW approximation and embedding operators; computational details for MD simulations and embedding models; numerical results, including ionization potential distributions for phenol and p-HBDI under different embedding schemes; summary tables of mean ionization energies, standard deviations, and comparison between semirigid and rigid MD geometries (PDF)

## #

Departamento de Física Teórica de la Materia Condensada, Universidad Autónoma de Madrid, E-28049 Madrid, Spain
