# Supplementary material for: GW Approximation Coupled with Classical Fluctuating Charges and Dipoles
Source: J Chem Theory Comput. 2025 Dec 23;22(1):371–84. doi: 10.1021/acs.jctc.5c01722 (PMC12805519; doi:10.1021/acs.jctc.5c01722)
Supplement: Supplementary file 1 [file ct5c01722_si_001.pdf]

Supporting Information for:
GW approximation coupled with classical fluctuating charges and dipoles

Giovanni Nottoli,[1] Piero Lafiosca,[1] Frank Ernesto Quintela Rodríguez,[1,*] Franco Egidi,[2] Arno Förster,[3] and Chiara Cappelli[1,†]

[1] *Scuola Normale Superiore*
Piazza dei Cavalieri 7 Pisa 56126 Italy
[2] *Software for Chemistry and Materials NV*
De Boelelaan 1109 1081HV Amsterdam The Netherlands
[3] *Vrije Universiteit Amsterdam*
De Boelelaan 1105 1081HV Amsterdam The Netherlands

I. GWA IN A NUTSHELL

To obtain GWA, we need to introduce some key physical quantities. Understanding the physical meaning of these operators allows us to develop a set of self-consistent equations, which, upon convergence, accurately describe the system.

- **Green's function** – G: The Green's function of a system is an essential operator that contains all the information about the behavior of electrons.[1–3] From this function, we can extract the spectral properties of our system as well as its dynamics. This function is defined in the complex domain of energies, the poles of this function within this domain are the energies of our quasiparticles.[1–3]

- **Polarizability** – χ: This quantity is a measure of the system's ability to become polarized, altering its electronic distribution in response to an external field.[1–3]

- **Screened Coulomb interaction** – W: While working with charged particles, the Coulomb operator v is the fundamental interaction between them. The Coulomb screened interaction W accounts for the effects of electron-electron interactions in a material, describing how the medium screens the interaction. In other words, W incorporates the dynamic response of the electrons to perturbations, effectively reducing the strength of the electron-electron repulsion by accounting for polarization effects in the material.[1–3]

- **Self-Energy** – Σ: it is the total energy of an electron in free space, isolated from other particles or photons, reflecting the energy changes induced in its surroundings by its presence. It contributes to the electron's energy or effective mass due to this interaction [4, 5].

* Current adress: Departamento de Física Teórica de la Materia Condensada, Universidad Autónoma de Madrid, E-28049 Madrid, Spain
† Email: chiara.cappelli@sns.it

In GWA, these quantities are interconnected, each influencing and defining the behavior of the others within a system of equations, formally called Hedin equations:[2, 6, 7]

$$G(1,2) = G_0(1,2) + \int d3d4\, G_0(1,3)\Sigma(3,4)G(4,2) \quad (1)$$

$$\chi(1,2) = -iG(1,2)G(2,1) \quad (2)$$

$$W(1,2) = v(1,2) + \int d3d4\, v(1,3)\chi(3,4)W(4,2) \quad (3)$$

$$\Sigma(1,2) = \Sigma^H(1,2) + iG(1,2)W(2,1) \quad (4)$$

In the above equations, the numbers are used to denote combined space and time variables for each electron. G_0 is called the *free* Green's function and it is calculated starting from the KS single-particle states, while G is the Green's function in which correlation effects have been taken into account employing GWA. In case of calculation on molecular system, the self-energy Σ can be partitioned into diverse contributions related to the portions of the molecular Hamiltonian, as shown in 4. Therefore, Σ^H in 4 is the contribution to the self-energy arising from the kinetic, ionic, and Hartree operators. Eqs. (1) and (4) are also known as the Dyson equations for the Green's function G for the self-energy Σ, respectively. This set of self-consistent equations constitutes the foundation of the GWA method. Once this set of equations is solved, it is possible to determine the self-energy in a many-body quantum system, from which we can correct the quasiparticle energies to account for electron correlation.

II. COMPUTATIONAL DETAILS

A. Gas-phase reference calculations

The vacuum ionization energies reported in the main text were obtained from single-point G_0W_0 calculations performed on gas-phase minimum-energy geometries. For both phenol and p-HBDI, the ground-state structures were optimized in vacuum at the PBE0/QZ4P level using ADF (no symmetry, default numerical settings). The optimized neutral (phenol) and anionic (p-HBDI) structures were then used as input for the G_0W_0 calculations,

which were carried out with the same protocol adopted for the solvated systems (Corr/TZ3P) and Corr/QZ6P basis sets and two-point CBS extrapolation).

B. QM/MM Calculations

Phenol and the GFP chromophore (p-HBDI) were initially optimized and characterized as minima on the potential energy surface (PES) at the B3LYP/6-311++G(d,p) level of theory, including the solvent effects of water through the Polarizable Continuum Model (PCM).[8]

Classical molecular dynamics (MD) simulations were carried out using the GROMACS2020 package. The solute parameters were generated using ACPYPE and ANTECHAMBER with the GAFF force field and CM5 charges derived at the B3LYP/6-311++G(d,p) level of theory.[9] Virtual sites were added to describe the lone pairs on oxygen atoms placed according to the Boys localization procedure [10]. Each solute was embedded in a cubic box of about 5 nm containing approximately 7370 water molecules for p-HBDI and 5600 water molecules for phenol, with water molecules described by the TIP5P model.[11] Planarity of phenol was maintained through dihedral restraints with a force constant of 10^3 kJ mol^{-1} rad^{-2}. For p-HBDI, two distinct simulations were performed: (i) a *rigid* model, where positional restraints were applied to heavy atoms in all directions with a strong force constant of 10^5 kJ mol^{-1} nm^{-2}, and (ii) a *semi-rigid* model, where the conjugated region was constrained to planarity through dihedral restraints of 10^3 kJ mol^{-1} rad^{-2}.

Energy minimization was performed using the steepest-descent algorithm. Two equilibration stages preceded the production runs. In the first stage (1 ns), the systems were gradually heated to 298.15 K in the NVT ensemble, using a 2 fs integration time step and the Velocity-rescaling thermostat (τ = 0.1 ps, Δt = 1 fs).[12] In the second stage (2 ns), NPT equilibration was conducted with a 2 fs time step under periodic boundary conditions. Pressure coupling was achieved using the Parrinello–Rahman barostat (τ_p = 2 ps, for phenol and semi-rigid p-HBDI) or the Berendsen barostat (τ_p = 2 ps, for rigid p-HBDI), with reference pressure of 1.0 bar.

Following equilibration, production MD simulations of 30 ns were performed for each solute–water system. The phenol and semi-rigid p-HBDI systems were simulated in the NPT ensemble, whereas the rigid p-HBDI system was evolved in the NVT ensemble. All simulations employed a 2 fs integration time step. The LINCS algorithm was used to constrain all bonds involving hydrogen atoms.[13] Long-range electrostatic interactions were treated using the particle–mesh Ewald (PME) method[14] with a grid spacing of 0.16 nm, cubic interpolation, and a Coulomb cut-off of 0.9 nm.

From each trajectory, 200 uncorrelated snapshots were extracted. For each snapshot, a solute-centered spherical cluster was generated with a radius of 18 Å for phenol and 20 Å for p-HBDI.

III. NUMERICAL RESULTS

Table S1. Computed mean ionization energies and standard deviations of phenol using different embedding approaches.

Model	Mean (eV)	σ (eV)
EE	8.147	0.374
DRF1	8.243	0.341
DRF2	8.132	0.330
DRF3	8.143	0.332
FQ1	8.387	0.263
FQ2	8.521	0.313
FQ3	8.653	0.436
FQFμ	7.422	0.495

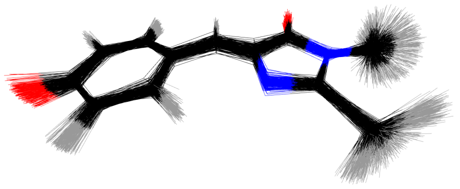

Figure S1. Superposition of MD snapshots for phbdi obtained with improper dihedral restraints. In this setup, strong improper dihedrals were applied in order to preserve the aromatic framework.

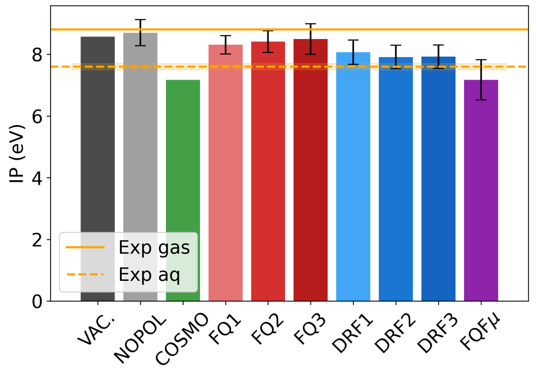

Figure S2. IP of phenol in aqueous solution computed on 200 uncorrelated snapshots.Values are obtained through extrapolation at a complete basis set based on Corr/TZ3P and Corr/QZ6P values. Bars indicate standard deviations. Horizontal orange lines correspond to the experimental IP in the gas phase (solid) and in water (dashed) [15].

The ionization potential is computed in aqueous solution and compared to available experimental

Table S2. Computed mean ionization energies and standard deviations of p-HBDI using different embedding approaches. Semi-rigid MD geometry

**Model**	**Mean (eV)**	**σ (eV)**
EE	5.89	0.68
DRF1	6.10	0.69
DRF2	5.64	0.61
DRF3	5.66	0.61
FQ1	5.18	0.46
FQ2	5.81	0.57
FQ3	7.44	0.83
FQFμ	7.13	1.04

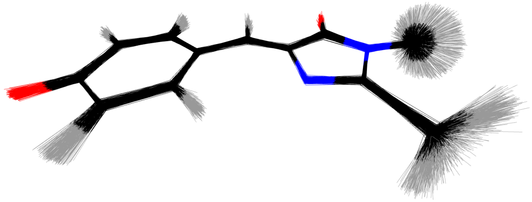

Figure S3. Superposition of MD snapshots for phbdi obtained with positional restraints. In this setup, GROMACS position restraints (`posre.itp`) were applied to all heavy atoms, effectively fixing the molecular scaffold and leaving only the methyl groups free to rotate.

Table S3. Computed mean ionization energies and standard deviations of p-HBDI using different embedding approaches. Rigid MD geometry

**Model**	**Mean (eV)**	**σ (eV)**
EE	6.01	0.72
DRF1	6.17	0.67
DRF2	5.71	0.59
DRF3	5.73	0.60
FQ1	5.28	0.48
FQ2	5.90	0.61
FQ3	7.53	0.88
FQFμ	7.23	1.08

estimates[15].

Table S4. Comparison of mean ionization energies (eV) obtained with semi-rigid and rigid MD geometries for selected embedding models.

Model	Flexible	Rigid	Δ (Rigid–Flex)
EE	5.89	6.01	+0.12
DRF1	6.10	6.17	+0.07
DRF2	5.64	5.71	+0.07
DRF3	5.66	5.73	+0.07
FQ1	5.18	5.28	+0.10
FQ2	5.81	5.90	+0.09
FQ3	7.44	7.53	+0.09
FQFμ	7.13	7.23	+0.10

As shown in Table S4, the ionization energies computed with semi-rigid and rigid MD geometries are in very close agreement. Across all models, the rigid geometry leads to slightly larger mean values, with systematic shifts of less than 0.1 eV. These discrepancies are therefore quantitatively minor when compared with the intrinsic uncertainties of the electronic structure calculations, and do not affect the overall qualitative picture. The consistent, though small, upward shift observed in the rigid case suggests that the lack of conformational relaxation of the solute leads to marginally stronger effective interactions with the solvent environment. Overall, the choice between rigid and semi-rigid geometries does not qualitatively alter the conclusions, but highlights the stabilizing effect of conformational semi-rigid on the computed ionization energies.

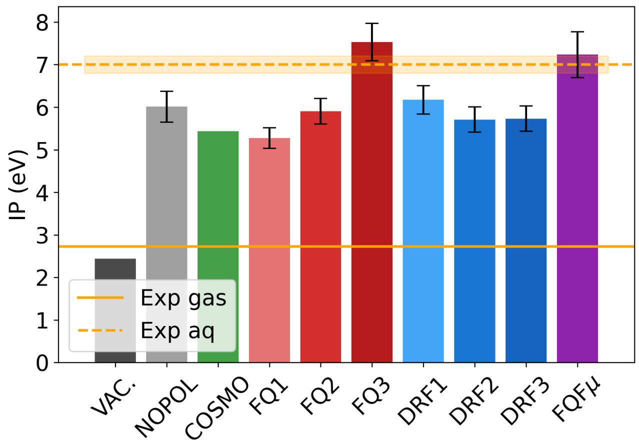

Figure S4. Distribution of computed ionization potentials (HOMO) for P-HBDI in aqueous solution, obtained from 200 snapshots of the rigid MD using different solvation models. Horizontal lines represent experimental reference values. The error bars indicate the standard deviation of the IP distribution for each model calaulated as reported.

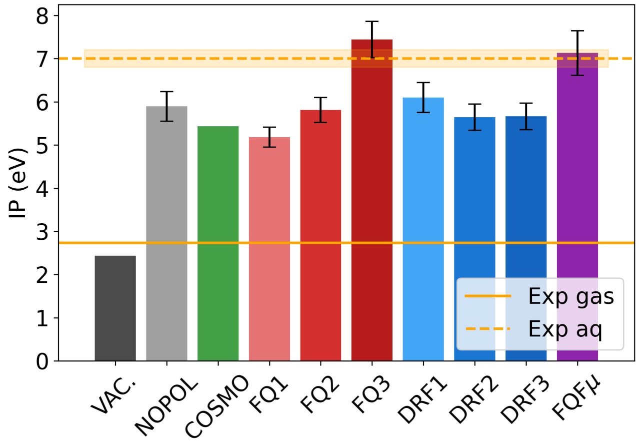

Figure S5. Distribution of computed ionization potentials (HOMO) for P-HBDI in aqueous solution, obtained from 200 snapshots of the semi-rigid MD using different solvation models. Horizontal lines represent experimental reference values. The error bars indicate the standard deviation of the IP distribution for each model calaulated as reported.

[1] R. D. Mattuck, *A guide to Feynman diagrams in the many-body problem*, 2nd ed., Dover books on physics and chemistry (Dover Publications, New York, 1992).

[2] L. Hedin, Physical Review **139**, A796 (1965).

[3] A. L. Fetter and J. D. Walecka, *Quantum theory of many-particle systems*, corr. repr ed. (Dover Publ, Mineola, N.Y, 2003).

[4] V. F. Weisskopf, Physical Review **56**, 72 (1939).

[5] K. Johnson, M. Baker, and R. Willey, Physical Review **136**, B1111 (1964).

[6] F. Aryasetiawan and O. Gunnarsson, Reports on Progress in Physics **61**, 237 (1998).

[7] A. Kutepov, V. Oudovenko, and G. Kotliar, Computer Physics Communications **219**, 407 (2017).

[8] J. Tomasi, B. Mennucci, and R. Cammi, Chemical Reviews **105**, 2999 (2005).

[9] J. Wang, R. M. Wolf, J. W. Caldwell, P. A. Kollman, and D. A. Case, Journal of Computational Chemistry **25**, 1157 (2004).

[10] S. F. Boys, Reviews of Modern Physics **32**, 296 (1960).

[11] T. J. Dick and J. D. Madura, in *Annual Reports in Computational Chemistry*, Vol. 1 (Elsevier, 2005) pp. 59–74.

[12] G. Bussi, D. Donadio, and M. Parrinello, The Journal of Chemical Physics **126**, 014101 (2007).

[13] B. Hess, H. Bekker, H. J. C. Berendsen, and J. G. E. M. Fraaije, Journal of Computational Chemistry **18**, 1463 (1997).

[14] T. Darden, D. York, and L. Pedersen, The Journal of Chemical Physics **98**, 10089 (1993).

[15] O. Tau, A. Henley, A. N. Boichenko, N. N. Kleshchina, R. Riley, B. Wang, D. Winning, R. Lewin, I. P. Parkin, J. M. Ward, H. C. Hailes, A. V. Bochenkova, and H. H. Fielding, Nature Communications **13**, 507 (2022).
